# Supplementary material for: Are carbon emissions trading and green financial instruments synergistic? -Comprehensive quantitative research based on content analysis
Source: PLoS One. 2024 Mar 7;19(3):e0298601. doi: 10.1371/journal.pone.0298601 (PMC10919601; doi:10.1371/journal.pone.0298601)
Supplement: S1 Data — (ZIP) [file pone.0298601.s001.zip › Data/地级市Z_F工作报告.zip!/╣π╢1⁄2/╣π╓▌╩╨/└┤╫╘╣1⁄2╓┌║┼í╛┬φ┐╦╩2╛▌═°í┐_2014.docx]

各位代表：

　　现在，我代表广州市人民政府，向大会作工作报告，请予审议，并请市政协委员和其他列席人员提出意见。

　　一、2013年工作回顾

　　过去一年，我们在市委的正确领导下，在市人大、市政协的监督支持下，认真贯彻党的十八大精神，坚持稳中求进的工作总基调，以新型城市化为引领，全力以赴稳增长、促转型、惠民生、增后劲，经济社会持续健康发展。全市地区生产总值15420.14亿元，增长11.6%；地方公共财政预算收入1141.79亿元，增长10.8%(按可比口径增长14.9%)；万元生产总值能耗下降5%左右，节能减排任务全面完成；城市居民人均可支配收入42066元，增长10.5%；农村居民人均纯收入18887元，增长12.5%；城市居民消费价格指数涨幅为2.6%。全面完成了市十四届人大三次会议确定的目标任务。

　　一年来，主要做了以下工作：

　　(一)多措并举促进经济增长。面对复杂严峻的国内外经济形势，坚持扩大内需、稳定外需、改善营商环境，确保了经济平稳较快增长。

　　内需拉动明显增强。围绕重点产业和重大发展平台招商，拓展投融资渠道。举办“新广州·新商机”系列投资推介活动，吸引国内外优质项目进驻。落实鼓励民间投资政策措施，激发民间投资热情。全年完成固定资产投资4454.55亿元，增长18.5%。组织广货展销活动，扩大消费需求。拓宽新兴消费市场，汽车消费逐步回暖，时尚消费持续兴旺，网上消费增势迅猛。推进重点商圈升级改造，优化消费环境。全年社会消费品零售总额6882.85亿元，增长15.2%。

　　外需市场保持稳定。面对全球经济不景气的形势，认真落实促进外贸发展政策，优化企业服务机制，促进外经贸稳定发展。培育外贸新业态，开展跨境贸易电子商务服务试点和外商投资商业保理业试点，服务贸易和境外投资发展取得突破。全年商品出口总值628.06亿美元，增长6.6%；实际使用外资48.04亿美元，增长5%；服务贸易总额567亿美元，增长41%；服务外包业务增长22%；对外投资增长1.8倍。

　　营商环境持续改善。深入开展“暖区、暖企、暖项目”活动，为重点企业、重点项目提供政策咨询、审批代办、要素保障等全方位服务。基本完成政府性债务风险化解方案制定和国有资产清产核资工作，债务风险总体可控。出台进一步加快民营经济发展“1+9”政策文件，实施《广州市建设工程项目优化审批流程试行方案》，优化投资环境。试点实施工商登记制度改革，推进“两建”工作，完善“营改增”配套措施，减轻企业负担。广州三度被福布斯评为中国大陆最佳商业城市第一名。

　　(二)加快推进产业转型升级。始终坚持“质量第一”发展理念，大力优化产业结构，打造优质发展平台，提升科技含量，增强经济发展后劲。

　　产业结构明显优化。制定《广州市加快推进十大重点产业发展行动方案》，统筹规划产业发展。三次产业比重为1.5∶33.9∶64.6。第三产业实现增加值9963.89亿元，增长13.3%。现代服务业加快发展。新增一批小额贷款公司等民间金融机构，广州股权交易中心挂牌企业达558家，金融业增加值突破1100亿元。会展业持续活跃，展览面积超过800万平方米。总部经济不断壮大，新认定总部企业89家。旅游业稳步增长，总收入2202.39亿元，增长15.2%。工业发展稳中提质。全市规模以上工业总产值17310.24亿元，增长12.9%，其中高新技术产品产值占43%，汽车制造、石油化工、电子信息三大支柱产业总产值增长16.3%。广汽集团入选“世界500强”。民营企业和小微企业加快发展，规模以上工业总产值分别增长17.8%和17.1%。战略性新兴产业蓬勃发展，新认定11个战略性新兴产业基地。农业平稳发展，增加值228.87亿元，增长2.7%。新兴业态亮点纷呈，聚集52家产值超亿元的移动互联网企业，成功创建国家电子商务示范城市，电商企业交易额超万亿元。

　　平台建设取得进展。坚持把战略性发展平台作为产业转型升级的主引擎，构筑“2+3+9”平台体系。加大两个新城区和三个副中心投资强度，重点推进市政交通、教育文化、医疗卫生等基础设施向新城区延伸。南沙新区明珠湾起步区建设启动，蕉门河中心区建设加快推进。中新知识城吸引近百个高端项目进驻，科学城功能进一步完善，生物岛建设加快推进。花都中轴线北部启动征拆，从化新城起步区完成征地工作，增城挂绿新城建设加速推进。国际金融城开工建设，海珠生态城启动十大项目，天河智慧城核心区土地征收基本完成，南站商务区核心区地下空间试验段动工建设，花地生态城电子商务集聚发展，国际健康产业城、空港经济区、国际创新城、黄埔临港商务区等重大平台建设按计划推进。

　　自主创新能力增强。实施《广州市科技创新促进条例》，创建校地协同创新联盟，创新机制日趋完善。成功举办第十六届留交会。国家超级计算广州中心“天河二号”运算速度世界第一。华南新药创制中心、华南干细胞与再生医学研究中心建设进展顺利。新增3家国家重点实验室和国家工程中心。全力创建全国质量强市示范城市，新增1家国家质检中心。“广州光谷”建设重启。建成科技企业孵化器66家，孵化面积410万平方米。全市专利申请量达39751件，其中发明专利12156件、增长23.8%。

　　(三)强化国家中心城市功能。以加快城市基础设施等功能建设为着力点，把优化环境作为增强城市综合竞争力的重要途径，不断提升国家中心城市的辐射力和影响力。

　　城市空间布局优化。推进“123”城市功能布局规划，新一轮城市总体规划已报国务院审批，完成平台核心区控制性详细规划，编制22个功能片区土地利用总体规划，推进1142个村庄规划编制，构建了新型城市化规划体系。“三规合一”成效显著，盘活存量建设用地规模128.8平方公里。开展节约集约用地量化评价，实施城市生态用地差别化管理，亿元GDP消耗土地减至0.83公顷，比2011年下降58%。开展国土管理改革试点，节约建设用地80多平方公里，拓展了发展空间。认真实施《珠江三角洲地区改革发展规划纲要》，获省委、省政府“四年大发展”考核验收第一名。

　　基础设施加快建设。新一轮总投资3000多亿元的交通基础设施建设全面展开，基本完成新建或在建的284公里地铁、208公里高速公路征地、借地和拆迁任务。推进白云机场扩建工程，第三跑道和第二航站楼开工建设，开展机场噪音区治理,白云机场旅客吞吐量突破5200万人次，居世界前10位。加快建设南沙港区三期工程，广州港口完成货物吞吐量4.55亿吨、集装箱吞吐量1531万标准箱，居世界前列。加快推进贵广南广铁路、穗莞深城际铁路建设，广州火车北站、南站和新塘站等综合交通枢纽加紧推进。地铁6号线首期开通运营，在建的6条地铁进展顺利，新建的7条地铁前期工作全面展开,海珠环岛新型有轨电车试验段开工建设。海珠桥维修工程完工。凤凰山隧道、花城大道东延线、猎德大道北延线等项目有序建设。新投放公交车500台和出租车1500台，新开通公交线路50条，新建公交专用道50公里，优化开通新水巴航线12条，完成25个交通拥堵点治理，立体交通网络进一步完善。电网工程、天然气利用工程顺利推进。儿童公园、儿童医院、老人院、福利院等惠民设施加快建设，176家社区金融服务站顺利完工。南汉二陵博物馆、大小马站书院街、南粤先贤馆、海事博物馆等建设全面展开，文化设施日趋完善。历史城建项目工程结算扎实推进。

　　生态建设全面推进。实施花城绿城水城规划，新建成300公里绿道和156公里景观林带，3个岭南花园和2个森林公园对外开放，完成19条高快速路、国省道的95个出入口和7条铁路景观整治。广州荣获全国“十大绿色建筑标杆城市”称号。加快城乡污水处理设施建设，城镇和农村生活污水处理率稳步提高；石井河截污、猎德涌改造、荔枝湾涌三期、东濠涌二期等河涌治理工程顺利推进，番禺金山湖、花都湖一期、南沙湿地二期建成开放，增城挂绿湖、天河智慧东湖、凤凰湖、广州北江引水、北部水厂一期、琶洲湾公共沙滩泳场、西郊沙滩泳场二期、海珠湿地二期、广州水博苑等工程加快建设。增加天然气、电力等清洁能源供应量，天河区建成无燃煤区。推进空气污染综合防治10大行动，控制机动车尾气、餐饮业油烟、挥发性有机物和扬尘污染，环境空气质量达标天数260天，比上年增加4天。

　　城市更新管理加强。按照“政府主导、利益共享、储备优先、成片开发”的原则，推进“三旧”改造，审批19个改造项目，总面积1.82平方公里，新增经营性用地和公益性设施用地1.4平方公里。“幸福同德围”、“美丽金沙洲”建设取得进展。115项烂尾楼、烂尾地、城市建设历史遗留项目基本得到解决。完成农村泥砖危房改造10118户和城市零散危房改造9.88万平方米。加大查处违法建设力度，拆除违法建设140万平方米、违法户外广告招牌12万多平方米，查处违法施工1926宗。推进新一轮314个幸福社区创建工作，推广越秀区社区网格化服务管理经验，加强黄埔区“综合化、网格化、信息化”服务管理平台建设，提升了城市管理水平。

　　(四)推进民生社会事业发展。坚持民生为先、底线思维，努力使发展成果惠及全体市民。市本级用于民生和各项公共事业经费462.3亿元，占公共财政预算支出的76.2%。全力兑现十件民生实事，医疗卫生、养老保险、住房保障、食品安全、文化惠民等事项超额完成。破解垃圾围城取得进展，一批收集、运输、处理、综合利用设施设备投入使用，资源热力电厂规划建设取得突破。

　　民生保障水平提高。着力保障就业，市财政投入专项资金4.6亿元，帮扶22.09万失业人员实现再就业，城镇新增就业27.75万人，城镇登记失业率为2.15%。提高企业离退休人员基本养老金和失业保险待遇，城镇居民医保、新农合政府资助标准每年320元以上，社会保险参保人数2913万人次，比上年增加近130万人次。城乡居民社会养老保险基础养老金从每月130元提高到150元。新增申领社会保障卡440万张。全市城镇最低生活保障标准提高到540元，农村平均低保标准提高到505元，“五保”供养标准提高到当地农村人均纯收入的70%，为20万困难群众购买重大疾病商业医疗保险。动工兴建1.2万张养老床位，新建成40个日间托老机构。落实中央和省房地产调控措施，筹建保障性住房18110套，新增发放住房租赁补贴3418户。设立来穗人员服务管理机构，提升服务水平。推进平安广州建设，深化社会治安立体防控，积极排查化解矛盾纠纷，全市受理刑事、治安案件类警情同比下降8.1%，广州荣获全国综治工作“长安杯”称号。全面推进“食得放心”城市建设，严厉打击食品药品违法犯罪，立案查处食品药品违法案件4672宗，破获食品药品类犯罪案件657宗。加强安全生产监督检查，企业安全生产基础进一步强化。

　　社会事业取得进展。加快教育综合改革。推动学前教育公益性普惠性发展，公办幼儿园占比超过30%，普惠性民办幼儿园占比超过50%。推动全市优质教育资源均衡布局，公办义务教育规范化学校达1028所，覆盖率为99.9%。大学城提升计划进展顺利，广州教育城一期加快建设。开展新一轮文化遗产普查工作，广州海上丝绸之路史迹“申遗”、重点文物修缮工作抓紧推进。举办广州艺术节、第六届中国国际漫画节、第九届中国音乐金钟奖等大型文化活动，成功创建全国版权示范城市，全国城市文明程度指数测评取得好成绩。免费向城乡居民提供11类37项基本公共卫生服务项目，社区卫生服务中心(站)实现街道全覆盖，农村三级医疗卫生服务网络建成。开通全市统一的“12320”卫生公益热线，广州地区 38 家大型医疗机构接入统一预约挂号系统。优生优育和计划生育服务不断优化。新建一批体育设施，公共体育场馆免费和优惠人数约550万人。广州职业足球夺得亚冠联赛冠军并蝉联中超冠军。举办世界羽毛球锦标赛等国内外重大赛事。与温哥华、伯明翰、里昂、布里斯托尔、法兰克福、汉班托塔等友城交流与合作取得新进展，我市行政首长连续3届当选世界城市和地方政府组织联合主席。

　　城乡一体扎实推进。规划建设8个现代农业园区，成立农业龙头企业协会，发展农民专业合作社830家。建立“菜篮子”最低保有量制度，建设3个蔬菜标准园、5个健康水产养殖示范场、4个年出栏10万头以上养猪场，国家华南亚热带良种奶牛繁育中心落户从化。市、区财政投入7.5亿元建设14个美丽乡村。加强农村土地综合整治和耕地保护，建设高标准基本农田20.9万亩，实现全市集体土地登记全覆盖。在430个贫困村开展新一轮扶贫开发工作。农村集体经济股份合作制改革取得新进展，农村基层公共服务站、“三资”交易平台建设基本完成。

　　各项事业全面进步。全民普法、法律援助工作进一步加强。梅州、清远、湛江新一轮扶贫开发“双到”工作全面启动。对口支援帮扶新疆疏附、西藏波密、贵州黔南等地区成效明显。国防动员、打私和海防、双拥和优抚安置、民族宗教、司法行政、妇女儿童、残疾人、慈善、侨务、对台、贸促、口岸、应急、气象、地震、民防、审计、统计、参事、文史、档案、保密、修志、仲裁、红十字会等工作取得新进步。

　　(五)加强政府自身建设。围绕建设人民满意政府，深化行政管理改革，大力转变工作作风，行政效率进一步提升。

　　办好议案决议和建议提案。坚持定期向市人大及其常委会报告工作，向市政协通报情况。认真执行人大议案决议，高度重视人大代表建议、政协提案办理工作，全年办理1005件，办理质量不断提高。继续推进2012年两件议案决议的办理落实，加快“珠江黄金岸线”建设和城市废弃物处置利用工作。认真落实市人大2013年1号议案决议，制订《广州市农转居人员基本养老办法(试行)》。

　　着力建设法治政府。推进行政立法工作，完成地方性法规草案5项，审议通过政府规章9项和规范性文件30项，清理行政处罚职权3138项、行政审批职权391项，办理行政复议1112宗。推进3705项市本级行政权力清单公开。坚持重大事项集体决策，市政府召开全体会议4次、常务会议48次、市长办公会78次。在全国首创市政府新闻发布会网络直播和市规委会议事现场直播，建立重大民生决策公众咨询监督制度。开展依法行政考核，加大对政府合同的审查力度。加强复议规范化建设，强化行政层级监督。广州分别被《中国法治政府评估报告2013》、《中国城市公共服务能力评估报告》评为第一名。130户市属国有企业全部纳入市国资委统一监管，事业单位清理规范和分类工作基本完成。

　　大力转变政府作风。扎实开展党的群众路线教育实践活动，坚决落实中央“八项规定”。全市性会议比上年减少37.5%，市政府、市政府办公厅发文比上年减少16.7%，考核检查评比表彰项目减少71%。设立机关作风“曝光台”、“回访台”和“排行榜”，在10个民生部门开展公共服务廉洁工作，促进政风行风好转。坚持厉行节约，严格预算审查制度，率先在全国实现三级政府“三公”经费信息公开，压减部门公用经费5%、经常性专项经费30%，努力建设阳光政府。

　　各位代表，过去一年取得的成绩来之不易。这是党中央、国务院和省委、省政府及市委总揽全局、正确领导的结果，是全市人民团结拼搏、不懈努力的结果，是社会各界人士和衷共济、鼎力支持的结果。在此，我代表市人民政府，向全市人民、驻穗部队官兵、各民主党派、人民团体以及社会各界人士表示崇高的敬意!向关心支持广州发展的港澳台同胞、海外侨胞、国际友人表示衷心的感谢!

　　我们清醒认识到，我市经济社会发展中还存在不少矛盾和问题。经济增长的新亮点不多，下行压力依然存在。产业层次不高，创新驱动、内生增长动力不足，部分企业生产经营困难，资源环境约束趋紧，转变经济发展方式的任务艰巨。城乡基础设施承载力不适应可持续发展需要，交通拥堵、垃圾围城、环境污染、安全生产风险等问题仍然突出。就业、社会保障、住房、教育、医疗、食品安全、社会治安等民生工作离群众期望还有差距。城乡之间和城区两个二元结构矛盾依然存在，农村生产生活条件有待进一步改善。政府转变职能不够到位，一些工作人员履职能力、勤政廉政意识和依法行政水平有待进一步提升。我们务必引起高度重视，采取切实有效措施，认真加以解决。

　　二、2014年工作安排

　　今年是贯彻落实党的十八届三中全会精神、全面深化改革的第一年，也是深入推进新型城市化发展的重要一年。世界经济缓慢复苏，但前景仍然充满不确定性。随着改革的不断深入，国内经济政策利好增多，有利于我市产业转型升级和长远发展。我们要抓住机遇，迎接挑战，改革攻坚，砥砺奋进。

　　今年政府工作的总体要求是：坚持以中国特色社会主义理论为指导，全面贯彻落实党的十八届三中全会和中央经济工作会议精神，坚持稳中求进、改革创新的核心要求，以推进新型城市化发展为引领，增创发展新优势，抢占经济制高点，全力以赴稳增长、调结构、促转型、惠民生、增后劲。更加注重全面深化改革，更加注重提高经济发展质量，更加注重提升城市功能，更加注重改善生态环境，更加注重创新社会治理，更加注重办好民生实事。坚持为民，用心做事，认真落实100项重点工作和100项重点工程，促进经济社会持续健康发展，建设低碳、智慧、幸福广州。

　　今年经济社会发展主要预期目标是：地区生产总值增长10%；地方公共财政预算收入按可比口径增长10%；固定资产投资增长14%；社会消费品零售总额增长13%；商品出口总值增长3%；万元生产总值能耗下降3.5%，化学需氧量、氨氮、二氧化硫和氮氧化物排放总量分别下降1%、2%、1%和1.5%；城乡居民收入增长与经济增长基本同步；城市居民消费价格指数涨幅控制在3.5%左右。重点抓好六个方面工作：

　　(一)深入推进重要领域改革，增创体制机制新优势

　　全面实施《中共广州市委贯彻落实〈中共中央关于全面深化改革若干重大问题的决定〉的意见》，努力增创法治化国际化营商环境新优势、产业竞争新优势、城乡发展一体化新优势、社会发展新优势、文化发展新优势和生态城市建设新优势。

　　深化经济体制改革，大力发展混合经济。推进国有企业改革，健全“大国资”监管模式，优化国资布局，加快市属国有企业股权多元化，完善法人治理结构，增强国企活力和竞争力。深化财政体制改革，建立事权与支出责任相适应的制度，推行全口径预算管理，加强政府投资管理，完善市、区财政管理体制，强化政府性债务管理及风险预警机制，坚持增收节支还债建设并重。推进国家服务业综合改革试点。深化产权制度改革，发展综合性产权交易市场。深化地方金融机构改革，发展企业股权交易、金融资产交易、碳排放权交易和航运交易市场,推进企业改制上市，完善金融服务体系。

　　深化行政体制改革，强化政府公共服务。推进政府职能转变和机构改革，完善来穗人员服务管理体制和食品药品监管体制，整合卫生与计生、文化广电和新闻出版等机构。实施行政区划调整工作。改革和完善国家级开发区管理体制机制，深化事业单位分类改革，开展事业单位法人治理结构和法定机构试点。推进综合执法体制改革和国有林场管理体制改革。进一步简政放权，深化行政审批制度改革，优化行政审批流程，推进行政审批标准化建设。铺开工商登记制度改革，实施企业投资备案制，提高行政效率。

　　深化治理机制改革，推行政民协同善治。坚持问需于民、问政于民、问计于民、问效于民，推行公众咨询监督委员会制度。完善立体化社会治安防控体系，建立调处化解矛盾纠纷综合机制。推动食品药品安全社会共治。健全重大社会决策稳定风险评估体系。推动村居治理创新，推进幸福社区建设。推行社区网格化服务管理，健全居民代表议事制度和自治章程治理制度，推进社区居委会直选。完善“村改居”社区管理机制。强化村民自治制度，推动行政事务、自治事务和集体经济组织经营事务三分离。深化社会组织登记、培育、监管体制改革，健全政府购买社会服务评估体系。建立健全社区志愿服务常态机制。

　　学习先进城市经验，扩大开放合作领域。创新外商投资管理体制，争取服务业对外开放先行先试，推进中新知识城中欧区域政策合作试点。改革对外投资管理体制，放开企业和个人对外投资。推进外贸结构调整，培育外贸新业态，加快发展服务贸易。加强与友城交流合作，办好第二届“广州奖”和中国国际友城大会暨广州国际城市创新大会，提升广州国际影响力。完善穗港、穗澳合作机制，加快南沙新区粤港澳全面合作示范区建设。依托航空枢纽、高铁和高速公路，拓展桂、黔、湘、赣、闽腹地，推进珠江-西江经济带建设。发挥省会龙头作用，加大与周边城市的融合力度，加强与粤东西北地区的经济合作，深化广佛同城、广佛肇经济圈合作，加快推进广清一体化进程。加强与梅州、清远、新疆疏附对口帮扶工作，做好对湛江、西藏波密、贵州黔南等地区的挂钩扶贫工作。

　　(二)加快转变经济发展方式，提高经济发展质量

　　坚持扩大内需战略，激发市场主体活力。发挥投资对促进经济增长的关键作用，争取国家、省重大项目落户广州。大力推进136个重点项目建设，力争实现年度投资1090亿元。推进汽车新项目、液晶显示、电子商务、互联网、通信导航等一批优质产业项目建设。发挥市场在资源配置中的决定性作用，在投资领域探索设立负面清单，向社会资本全面开放。改善政务环境和营商环境，落实加快民营经济发展“1+9”政策文件，提供全方位政府公共服务，建立中小企业服务分中心或代办机构，遴选一批高成长性中小微企业纳入重点库，促进民营企业和中小微企业发展。鼓励广州股权交易中心为民营企业开展股权登记、托管、查询等服务，发展小额贷款公司、融资性担保公司和民营银行，缓解企业融资难问题。加快广州基金投资运作，运用市场化方式筹措资金。出台鼓励工业设计、融资租赁、健康服务、游艇业等新兴产业发展的政策措施，促进新兴产业蓬勃发展。优化消费环境，打造16个都会级商圈，提升16个区域级商圈功能，开拓“21世纪海上丝绸之路”通道，力促广货“中扩北上西进”。 认真做好第三次全国经济普查工作。推进社会信用体系建设，基本建成市级公共信息管理系统框架。

　　全力发展重点产业，突出重大平台招商。认真落实十大重点产业发展行动方案，全力发展汽车、精细化工、重大装备、新一代信息技术、生物医药、新材料、新能源与节能环保、商贸会展、金融保险、现代物流十大重点产业。加强对重点产业发展的支持引导和统筹协调，创新产业集群招商、联合攻关、聚集发展思路。加强平台招商，创新开发模式，实施分类扶持政策，引导重点产业向重大发展平台集聚，打造产业转型升级示范区。在“2+3+9”平台的基础上，增加北京路文化核心区和白云综合服务功能区。瞄准全球1000家行业龙头、国内1000家知名企业，建立招商引资目标库，争取更多的跨国公司、重大产业项目落户广州。加大资金、土地等政策扶持力度，加快十大重点产业的培育和发展，促进产业转型升级。加快汽车企业兼并重组和产业链整合，力争汽车制造业产值达到3800亿元。加快萝岗日化和黄埔石化产业基地、南沙中船海洋工程装备、番禺和花都南车轨道交通装备、白云机场飞机维修基地、工业机器人产业园建设。发挥国家超级计算广州中心的支撑作用，推动中国电信云计算中心和云安全技术公共服务平台建设。加快生物岛、广药白云基地一期和广州健康医疗中心建设。抓紧引进广州民营科技园新材料龙头企业。加强“城市矿产”开发利用，制定碳排放交易实施办法，加快太阳能和天然气分布式发电示范项目建设，推进国家循环经济示范城市创建工作。以专业批发市场为重点带动传统商贸业转型升级，推进大罗珠宝城、狮岭皮革皮具城、新塘牛仔城等升级改造。优化琶洲会展区规划设计。加快广州民间金融街三期建设。发展金融保险、电子商务、现代物流、工业设计、服务外包、时尚创意等高端服务业。推动白云机场综合保税区、南沙保税港区、广州保税区等海关特殊监管区域建设，创造条件申报自由贸易园区。

　　增强创新驱动能力，实施人才兴市战略。发挥政府引导作用，加大科技研发投入，提升R&D占比。改革科技服务与管理体制机制，发挥校地协同创新联盟的作用，建设一批协同创新平台。推进中乌巴顿焊接研究院等重大科技创新平台建设，力争国家重点实验室、工程中心、质检中心、技术中心和工程实验室等达到70个。突出企业在创新中的主体地位，引导企业加大科技研发投入，力争新增20家创新型企业和100家科技“小巨人”企业。启动实施科技企业孵化器倍增发展计划，争取孵化器数量达85家、面积500万平方米。加快中新知识城国家知识产权保护与服务综合改革试点工作。实施人才兴市战略，推进“创新创业领军人才百人计划”和“万名海外人才集聚工程”，推动高技能人才精工项目和人才资源服务产业园建设，构筑创新创业人才高地。以创建全国质量强市示范城市为抓手，实施名牌战略和标准化战略，以自主关键技术为核心参与国际标准的制修订，加快国家检验检测高技术服务业集聚区建设，进一步增强“广州质量”、“广州标准”的影响力。

　　(三)大力发展社会民生事业，善政惠民维护公平正义

　　对照先进城市标杆，全面发展社会事业。巩固提升华南地区教育、文化、卫生、体育中心地位。大力发展教育事业。加快创建“省推进教育现代化先进市”，促进学前教育健康规范发展，推动义务教育资源均衡优质发展，落实大学城提升计划，推进广州教育城建设，推行考试招生制度改革，促进各级各类教育协调发展。大力发展文化事业。开展全市文化遗产普查，做好文化名城保护工作，弘扬传承岭南文化。办好“一区一品牌”民俗文化活动。建设“四馆一园”等重点文化基础设施。加快华侨博物馆、粤剧博物馆、西关泮塘建设。试点建设街(镇)综合性文化服务中心，完成广州少年儿童图书馆改造。组织文艺院团送戏下基层。巩固文化体制改革成果，培育国有骨干文化单位和领军企业。鼓励社会资本参与公共文化建设，培育文化非营利组织。深化文明城市建设，巩固创建工作成果。大力发展卫生事业。加快市第八人民医院二期项目等医疗基础设施建设。推进公立医院改革试点。扶持民间资本发展医疗服务业，严厉打击非法行医，推进“平安医院”建设。完善落实基本药物和全科医生制度，加强基层卫生服务机构建设，逐步推行基层首诊，建立基层医生与居民契约服务关系。完善人口计生管理服务，推进母婴安康行动计划和优生促进工程，落实国家和省“单独二孩”政策。大力发展体育事业。发展群众体育、竞技体育和体育产业。推进创建全国安全发展示范城市，遏制重特大安全事故。加强物业管理和物业专项维修资金管理，推进小区业主委员会建设。开展“六五”普法，推进法律援助工作。加快气象现代化，强化气象为经济社会服务功能。继续做好民族宗教、国防动员、双拥、海防、打私、侨务、对台、应急、地震、民防、参事、文史、档案、保密、修志等工作。

　　完善社会保障体系，统筹确保底线民生。健全促进就业创业的政策体系，提升就业援助服务精细化水平，加强失业形势动态监测。扩大社会保险覆盖面，加大对困难群体参加社会保险的资助力度。建立城乡居民基础养老金正常调整机制，完善城乡居民养老保险缴费激励机制。加大养老机构建设统筹力度，实施入住公办养老机构轮候制度，对家庭经济困难老年人入住养老机构实行政府补贴，支持发展民办养老机构。实行医疗保险城乡统筹，建立城乡居民医疗保险制度。进一步完善重特大疾病救助制度。完善工伤和生育保险体系，促进外来务工人员参加工伤保险。完善和扩大对来穗人口的公共服务，落实异地务工人员积分入户政策。

　　统筹城乡一体发展，拓宽农民增收渠道。抓好现代农业园区建设，完善和落实科技兴农政策，鼓励农村金融创新，扶持农业龙头企业上市融资，引导农民专业合作社规范、快速发展，推进家庭农场注册登记，加快形成新型农业经营体系，完善农田水利基础设施，抓好5个万亩蔬菜产业化生产基地、10个蔬菜专业村、1个现代化奶牛场等市内“菜篮子”基地建设。推进市外“菜篮子”生产基地认定，加强农产品质量安全监管与动植物疫病防控。加强对农村土壤重金属污染治理。做好新一轮农村扶贫开发。继续做好农村土地确权登记工作，深化农村集体经济股份合作制改革，加强集体经济规范管理。推进集体建设用地使用权流转配套政策出台和流转试点工作。完善农民宅基地制度，健全农村产权流转交易市场，拓展农民财产性收入渠道。深化征地制度改革，建立和完善多元化征地补偿安置机制。推进解决从化、增城历史留用地欠账问题。

　　办好十件民生实事，坚持发展成果惠民。一是保持就业稳定。帮助16万名城镇登记失业人员实现再就业。举办300场次就业专场招聘会，组织5万名以上本市持培训券人员参加职业技能培训。实现全年城镇新增就业人数20万以上，广州生源应届高校毕业生就业率达90%以上，城镇登记失业率控制在3.5%以下。二是提高住房保障水平。新建和筹集保障性住房11000套，新增发放住房租赁补贴1300户。三是加强食堂食品安全监管。完成1000家中小学校(职业中专、技工院校)和幼托机构食堂食品安全视频监控系统建设。四是加快清洁能源改造。新增管道燃气用户30万户。完成1000台燃煤燃重油锅炉的淘汰或清洁能源改造。五是改善公交出行。新增投放公交运力500台、公交线路 25条、公交专用道 50 公里。六是整合政府非紧急类服务热线为12345。实现热线平台与政务服务中心实体大厅、网上办事大厅互联互通，打造“政务咨询、民生诉求、政民互动、投诉举报、效能监察”为一体的公共服务平台。七是改善老人、妇女和儿童福利条件。改造和新建 400 个农村老年人活动站点。免费为全市农村妇女提供宫颈癌、乳腺癌检查。推进“1+12”儿童公园建设，一半以上儿童公园(一期)建成开放。八是实施特殊教育普及和质量提升计划。研究开发特殊教育专用教材学材1套，建设随班就读指导中心2个、随班就读资源室24间。为各区12所特殊教育学校各配置特殊教育专用教学器具、仪器1套。实现残疾儿童义务阶段教育“零拒绝”。九是支持全民健身活动。建设30个小型足球场。推进36个街镇“一站两点”(文化体育工作站、国民体质监测点、社会体育指导员服务点)试点工作。公共体育设施免费和优惠时段分别由每周10小时增加到14小时。十是丰富群众文化生活。免费安排群众文化活动1500场、艺术进校园20场及发放惠民文艺演出、节展及展览等文化活动门票10万张。推进市广播电视无线覆盖工程建设，实现广州市广播、电视第一套节目在行政区域达到90%以上的覆盖率。

　　(四)加强城乡规划建设管理，调整完善城市功能

　　强化规划功能引领，节约集约利用土地。优化提升一个都会区、创新发展两个新城区、扩容提质三个副中心，推进城市总体规划、功能区控规的实施，应用“三规合一”成果，推进历史文化保护规划和村庄规划编制。加强土地用途管制和生态用地保护，扩大农村集体经营性建设用地权能。开展不动产统一登记，优化建设用地指标分配，严格落实产业用地标准，完善农村土地综合整治支持政策，扩大土地有偿使用范围，提高市场配置土地资源的能力，强化土地节约集约利用。推进城市更新，坚持与产业升级、土地节约集约和储备、住房保障、文物保护等工作相结合。完善和用足用好用活“三旧”改造与城市更新政策，集中开展成片“三旧”改造，加快推进国际金融城、广钢新城、大坦沙、黄埔临港商务区等项目建设。推进“城中村”改造，完善改造总体规划、工作方案和政策措施，推进“三线入地”，推进水、电、气、消防等公共基础设施城乡均等化。积极推进“珠江黄金岸线”建设，加快“幸福同德围”、“美丽金沙洲”建设，启动罗冲围综合整治，整体提升城市建设水平。加大执法力度，遏制违法建设。

　　强化交通功能引领，布局基础设施建设。加快推进白云机场第三跑道、第二航站楼建设和噪音区治理，开展商务机场前期工作。加快南沙港区集装箱三期、深水航道拓宽、散货码头等工程建设，推进南沙疏港铁路建设。完成贵广、南广铁路广州枢纽工程。加快地铁5条续建线路、6条新开工线路建设。完成海珠环岛新型有轨电车试验段工程。推进大广、广明、凤凰山隧道等一批高速公路项目建设；推进同德围南北高架路、洲头咀隧道、流花湖隧道、空港大道二期、花城大道东延线、猎德大道北延线、黄埔疏港道路、海珠环岛路等市政项目。加强城市交通综合治理，建成2个公交站场和100座公交候车亭，完成15个交通拥堵点整治，加强公交与地铁、BRT线路衔接。强化中心区与高快速路网、与周边城市无缝对接。推进3座50万伏变电站等电网工程落地，加快输气工程建设。

　　(五)继续改善城乡生态环境，建设宜居美丽生态广州

　　建设花城绿城水城，展现城更美水更清。加快岭南花园、森林公园、生态景观林带和碳汇林建设，新建绿道300公里。落实省政府水环境治理目标，开工整治石井河、流溪河、白坭河等19条广佛跨界河涌及市区部分河涌，开展对已整治河涌的复查和治理。完善城市防洪排水系统，建设东濠涌深层隧道试点工程。抓紧推进石榴岗河水闸、北部水厂一期、石井净水厂和广州北江引水等工程建设。完善城乡污水处理设施建设。完成天河智慧东湖、凤凰湖、增城挂绿湖、西郊沙滩泳场二期、琶洲湾公共沙滩泳场、海珠湿地二期、广州水博苑和长洲岛新担涌水闸工程建设。划定生态控制线，完善经济补偿机制。

　　改善环境空气质量，保障市民身体健康。按照珠三角大气环境治理要求，加强空气环境治理。全面使用国Ⅳ标准车用柴油，淘汰营运黄标车7000辆，高污染汽车限行范围扩大到528平方公里，严控冒黑烟和排气超标车辆上路，加强机动车排气污染执法检查。推进燃煤电厂技术改造。加快新能源利用设施建设。加大车辆使用新能源力度，发展电动汽车。强化对餐饮业油烟的排放控制和重点排污企业的监控，严肃查处违规排放企业。

　　推行垃圾分类处理，加快处理设施建设。推进垃圾源头减量、分类投放和分类收集运输。完成第二批垃圾压缩站升级改造，建设一批多功能中转站。推进生活垃圾计量收费试点，开展固体废弃物资源化利用和无害化处理试点。健全建筑废弃物管理信息平台，推进建筑废弃物前段源头减量、过程监督控制、后端循环利用的一体化综合管理。加快落实资源热力电厂、餐厨垃圾处理厂和填埋场等设施建设。

　　建设文明美丽乡村，改善农村生态环境。完成第二批27个市级美丽乡村试点创建和验收任务，开展第三批创建点选点。推进农村生活污水处理、农村环境综合整治和生态建设，加强农业面源污染防治，保障农村饮用水安全，推进农村生活垃圾收运处理管理体系建设。

　　(六)用心做事建设法治政府，坚持为民提供优质服务

　　坚持务实依法行政，建设阳光法治政府。认真执行市人大及其常委会各项决议和决定，落实重大决策之前向市人大常委会报告制度，切实做好人大代表建议和政协提案办理工作，定期通报办理情况。坚持依法、科学、民主决策，落实《广州市重大行政决策程序规定》，扩大公众对政府决策的有效参与。坚持依法办事，严格行政执法，提高行政执行力。自觉接受人大、政协和社会监督。

　　提高行政办事效率，建设勤政廉洁政府。强化政府在公共服务、市场监管、社会管理和环境保护方面的职能。加强政府应急体系建设。推进信息政府和电子政务建设，建立互联互通的信息共享体系。扩大行政决策公开领域和范围，探索推行执法投诉和执法结果公开制度。坚决贯彻中央“八项规定”等系列要求，切实转变机关作风。加强“三公”经费管理，压缩行政运行成本。深入推进政风行风建设，加强监督检查，整治庸懒散奢等不良风气。强化重大项目经济责任和绩效审计。加强预防和惩治腐败制度建设，开展廉政风险防控，规范行政权力公开运行，提高政府公信力。

　　各位代表，梦想创造奇迹，奋斗铸就辉煌。站在新的历史起点的广州，必须志存高远，奋发图强。让我们紧密团结在以习近平同志为总书记的党中央周围，在市委的坚强领导下，为实现广州人民的期待和梦想，为建设低碳、智慧、幸福广州而倍加努力工作!
